# Malignant Priapism, an Ominous Sign With Dismal Prognosis: A Case Report

**DOI:** 10.7759/cureus.80222

**Published:** 2025-03-07

**Authors:** Rajan Ravichandran, Roshan Reddy, Velmurugan Palaniyandi, Hariharasudhan Sekar, Sriram Krishnamoorthy

**Affiliations:** 1 Urology, Sri Ramachandra Institute of Higher Education and Research, Chennai, IND

**Keywords:** metastasis, penectomy, penile nodules, penis, priapism

## Abstract

Malignant priapism is a rare and serious manifestation that results from advanced malignancy. It is caused by the infiltration of tumour cells into the penile tissue. Genitourinary cancers are the most common cause, especially prostatic adenocarcinoma. It indicates widespread metastatic disease and is associated with a poorer prognosis. It presents as a prolonged, painful erection that is not related to sexual activity and is often refractory to conventional treatments. It is rare and usually diagnosed late in the disease course.

We report a 70-year-old male who underwent transurethral resection of the prostate (TURP) and bilateral orchidectomy for locally advanced prostatic adenocarcinoma and lost to follow-up. He presented six months later with painful priapism, multiple penile nodules, and acute urinary retention. Imaging studies, including high-resolution penile ultrasound and 18F-fluorodeoxyglucose positron emission tomography (18F-FDG PET) scan, confirmed multiple penile metastatic deposits.

Despite initial pain management with nonsteroidal anti-inflammatory drugs (NSAIDs) and opioids, his refractory symptoms necessitated a total penectomy with perineal urethrostomy. Histopathological examination confirmed infiltrating carcinomatous deposits. Unfortunately, despite aggressive management, the patient eventually succumbed to the disease.

This report emphasizes the aggressive character of penile metastases from prostate cancer, the challenges in diagnosis and treatment, and the need for a multidisciplinary approach. Early recognition and palliative strategies are essential for maximizing the quality of life.

## Introduction

Prostate carcinoma is the second most common carcinoma in men worldwide. It has great potential for metastasis. The most common sites of metastasis include bones, lymph nodes, lungs, and liver. Despite its rich vascularity and complex venous and lymphatic drainage, penile metastasis due to prostate carcinoma is rare and accounts for less than 1% of all secondary penile malignancies [1]. It has unique diagnostic and therapeutic challenges due to its rarity and often leads to delays in diagnosis. Penile metastasis indicates widespread disease and has a dismal prognosis. Routes of metastasis include either direct invasion from adjacent structures, hematogenous route, lymphatic spread, or iatrogenic implantation [2]. Extensive venous connections between the prostate and the penile vasculature, which consists of the prostatic venous plexus and the deep dorsal vein of the penis, are responsible for hematogenous metastasis. Lymphatic dissemination occurs through interconnected lymphatic channels. Penile metastasis presents clinically as a painful nodule, ulceration, or malignant priapism - a distressing rare condition of persistent, painful erection due to a tumor infiltrating into corpora cavernosa. Malignant priapism has a poor therapeutic outcome, often reflecting extensive vascular involvement. Penile metastases are frequently mistaken as a benign condition or primary penile malignancy, which complicates prompt treatment. Accurate diagnosis depends on a histopathological analysis of needle biopsy and immunohistochemistry staining for prostate-specific antigen (PSA) and prostate-specific membrane antigen (PSMA). Prostate cancer metastasis can be distinguished from other penile tumors with serum PSA, which is a valuable marker for both diagnosis and prognosis [3].

Treatment options include radiation therapy, androgen deprivation therapy, and radical penile amputation in selective cases. Metastatic prostate cancer with involvement of the penis and priapism is aggressive; hence, they are managed mostly palliatively, with the intent of enhancing the patient's quality of life rather than curative therapy. Being uncommon with varied clinical presentations, prompt diagnosis is essential for effectively managing such a scenario. Increased awareness among clinicians will help in early intervention, maximize palliative care, and improve the quality of life for impacted patients.

## Case presentation

An elderly male in his late 70s presented with voiding lower urinary tract symptoms and acute urinary retention. On clinical examination, the patient had a suspicious hard nodule in the prostate, and serum PSA was normal. He underwent a transrectal ultrasound (TRUS)-guided prostate biopsy. Histopathological examination revealed adenocarcinoma with neuroendocrine differentiation with a Gleason score of 5+5. PSMA PET was done, which showed a PET avid lesion in the prostate with extracapsular extension with no other PET avid lesion or distant metastasis. Treatment options were discussed. Considering the patient's age, the patient was initially subjected to hormonal therapy in the form of bilateral orchidectomy along with transurethral resection of the prostate (TURP) for his obstructive voiding and acute urinary retention. The patient voided post-catheter removal on postoperative day 5. He lost follow-up and presented to us in the emergency department six months later with swelling of the glans penis, persistent painful erection for three days, and inability to void for one day.

Clinical examination and diagnostic workup

**Figure 1:**
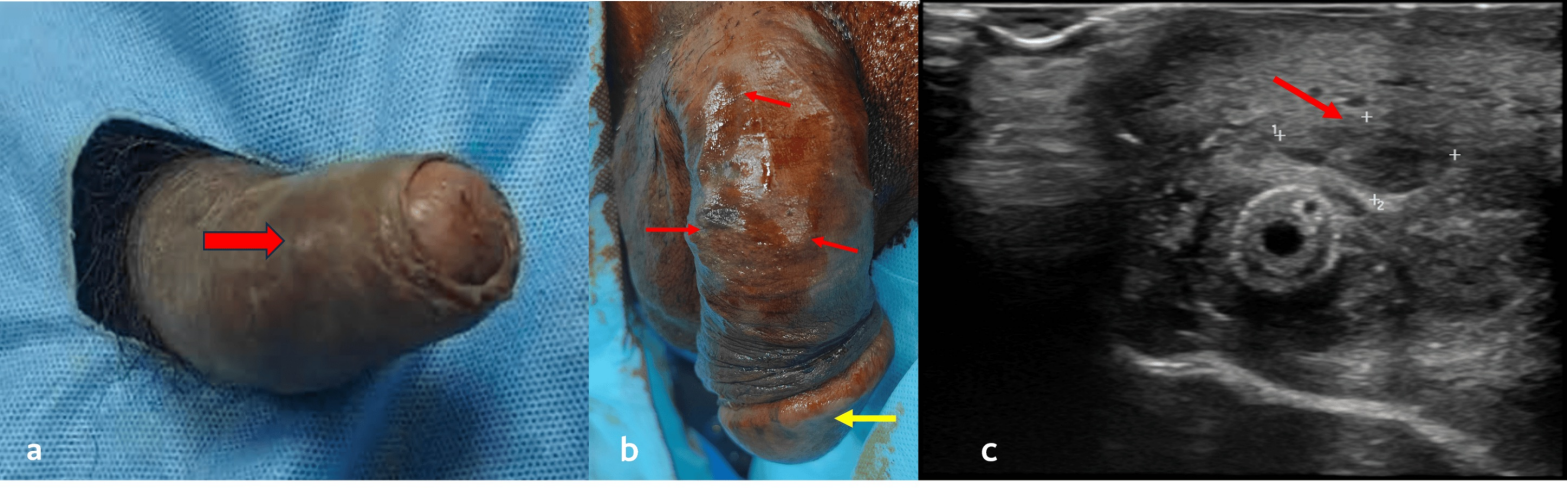
Clinical image (a and b) showing priapism and penile nodules, and ultrasonography (c) of the penis showing heteroechoic lesion Engorged penis with nodule over corpus cavernosum (arrow) (a), nodule over glans penis (yellow arrow), and multiple nodules over corpus cavernosum (red arrows) (b). Ultrasonography of the penis showing heterogeneous, predominantly hypoechoic lesion in the right corpus cavernosum (arrow) (c).

Clinical examination revealed multiple tender nodules over the glans and bilateral corpora cavernosum, and the penis appeared engorged (Figure 1a, 1b) with a painful palpable bladder suggestive of acute urinary retention. The patient was catheterized, and given the history of prostate carcinoma, a clinical diagnosis of penile metastasis was made. High-resolution ultrasound of the penis was done, which showed multiple heterogeneous predominantly hypoechoic lesions with increased peripheral vascularity in the bilateral corpora cavernosum, with the largest lesion measuring approximately 12 x 6 mm in the right corpora cavernosum, another lesion in the corpora spongiosum of roughly 7 x 7 mm present over its mid ventral part, another lesion in the left lateral aspect of glans measuring 12 x 8 mm, and in the dorsal aspect of glans measuring 10 x 6 mm (Figure 1c).

**Figure 2:**
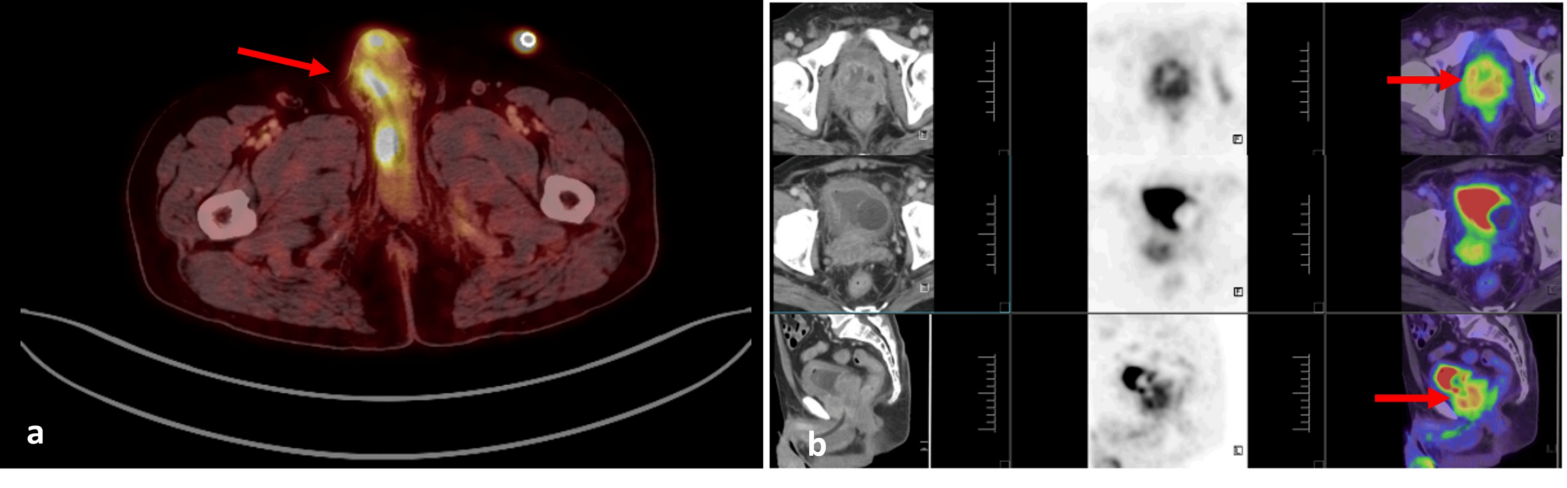
Positron emission tomography showing FDG avid penile lesions Multiple metabolically active FDG avid lesions in the penis (suggestive of metastasis) (a), malignant-looking mass lesion seen replacing the prostate and involving bilateral seminal vesicles and trigone of the urinary bladder (b).

These findings raised a strong suspicion of metastatic penile involvement. Whole body 18F- fluorodeoxyglucose (FDG) positron emission tomography (PET) was done to know the extent of the metastatic involvement, which showed multiple metabolically active FDG avid lesions in the penis, confirming the metastatic deposits (Figure 2). Laboratory evaluation revealed haemoglobin of 10.7 g/dL indicative of mild anaemia, leukocytosis (13.08 × 10^9^/L) with neutrophilia (80%), and a raised C-reactive protein (16 mg/dL), suggesting underlying systemic inflammation and serum PSA of 3 ng/ml. Urine analysis showed pus cells of 80/high power field and plenty of red blood cells suggestive of ongoing inflammation or infection in the urinary tract and was treated with empirical antibiotics.

Management approach

At first, the management goal was to relieve pain. The patient was started on nonsteroidal anti-inflammatory drugs (NSAIDs) but was not responding. Hence, opioids were added. Given the worsening pain and in view of multiple infiltrative penile metastatic nodules, it was decided to proceed with surgical intervention. A total penectomy with perineal urethrostomy was performed to provide symptom relief. The surgery was uneventful, and the patient tolerated the procedure well. The patient was symptomatically relieved of pain.

Histopathological findings

**Figure 3:**
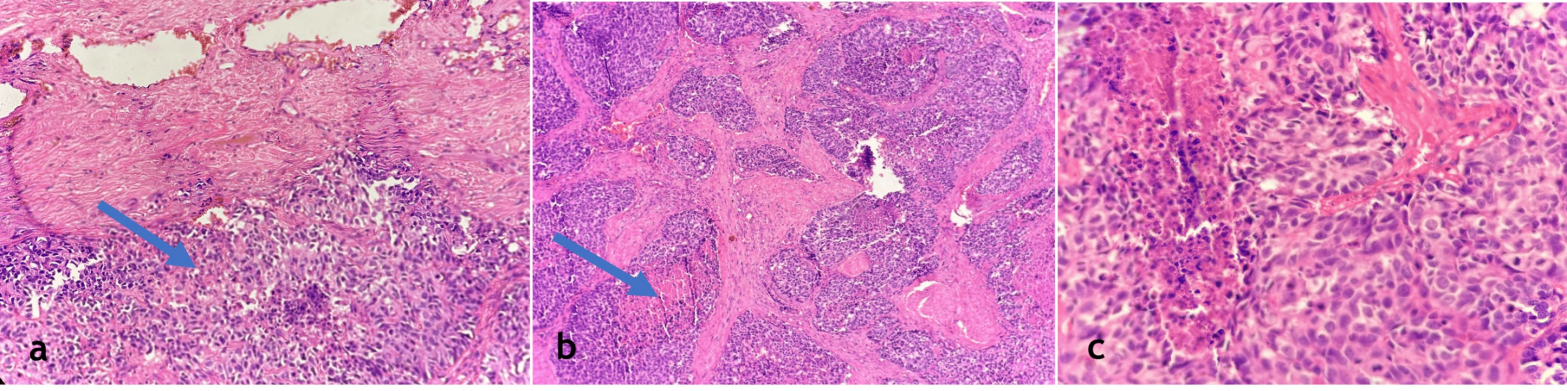
Histopathological examination with hematoxylin and eosin (H&E) showing tumor cells with atypical mitosis 200x magnification showing skin with an underlying malignant neoplastic lesion composed of tumor cells arranged in nests (arrow) (a), 200x showing tumor cells arranged in nests with comedo necrosis (arrow) (b), and 400x showing tumor cells that are round to oval with dispersed chromatin with atypical mitosis (c).

Histopathological examination of the excised penile tissue showed infiltrating malignant neoplasm with tumor cells arranged in nests and sheets with areas of comedo necrosis. The individual cells are round to oval with scant eosinophilic cytoplasm and round to oval nuclei with dispersed chromatin. Atypical mitosis is noted, confirming the aggressive nature of the metastatic deposits (Figure 3). These findings were consistent with infiltrating carcinomatous deposits, supporting the diagnosis of penile metastasis from primary prostate carcinoma.

Postoperative outcome and follow-up

The patient had significant symptom relief from the pain that was refractory to the medical management. He was discharged with prompt palliative care measures. The per urethral catheter was removed after two weeks, and the patient was voiding well. The patient was informed about the need for prompt oncological follow-up. Additional systemic therapies were considered as the disease was of advanced stage with metastatic involvement.

## Discussion

In 1961, Abeshouse detailed 140 cases of malignant priapism [4]. According to a review of the literature, metastatic priapism is a relatively uncommon first presentation of cancer, particularly when it comes to primary prostate adenocarcinoma. In 2006, Cherian et al. collected 372 case reports of this condition [5]. Despite its rich and intricate vascular circulation, penile metastases are not that common. A few physiological mechanisms, including the venous and lymphatic retrograde route, the arterial dispersion route, the direct extension, and the secondary to instrumentation route, were hypothesized by Abeshouse and Jacob Cherian to explain malignant priapism [4, 5].

Malignant priapism poses a significant clinical challenge. The prolonged and persistent penile erection is caused by malignant infiltration of tumor cells rather than vascular or neurological causes. Being rare, understanding the pathophysiology, presentation, and management is crucial both for early detection and for improving patient outcomes. Penile metastasis can originate from various malignancies like prostate, bladder, rectal, and renal carcinomas. Metastatic penile deposits are often a sign of an advanced disease stage and have a poor prognosis. It usually appears as several painless nodules on the penile skin's surface that mimic syphilitic lesions and have just begun to ulcerate. Primary penile tumors, syphilis, TB, and nonspecific inflammatory diseases must all be included in the differential diagnosis [4]. A careful and thorough examination and an appropriate diagnostic workup are mandatory for an accurate diagnosis and prompt management.

Penile ultrasonography is usually the quickest method for identifying metastasis and distinguishes high and low-flow priapism [6]. Histopathological studies, either biopsy or fine needle aspiration, remain the gold standard for definitive diagnosis.

Corpus cavernosal biopsy is the most commonly used technique to obtain a histological diagnosis [7]. Color Doppler ultrasound can assess the vascularity of the metastatic lesion in the corpora cavernosum or spongiosum. It typically exhibits vascularization and varied echogenicity [8]. Both magnetic resonance imaging (MRI) and computed tomography (CT) are other dependable alternative methods to assess the severity of an illness and to confirm the diagnosis [9]. Using a T1-weighted sequence, penile MRI can detect thrombosis and bleeding within the corpora and provide high-quality pictures of the penile tissue and the cavernosal smooth muscle. CT imaging is instrumental in staging the disease and identifying associated metastatic sites.

Metastatic penile lesions with priapism can be managed initially with conservative medical therapy and shunting for palliation of symptoms. If it fails, then in the event of persistent discomfort or obstructive urinary symptoms which was brought on by corpus cavernosal infiltration, aggressive interventions like local lesion excision, partial or complete penectomy, or both may be necessary depending upon the extent of the disease and the patient's overall condition.

Surgically unfit patients will benefit from chemotherapy and radiation therapy. It helps reduce the tumor burden, relieves symptoms, and potentially prolongs survival. However, given the aggressive nature of metastatic priapism, the prognosis remains poor, with most patients having significantly limited survival following diagnosis.

The primary tumor, its extent, the clinical condition of the patient, and the presence of metastatic disease determine the prognosis of the patient with malignant priapism. Several studies have evaluated the survival rates of patients with metastatic priapism. The survival rate for malignant priapism ranges from one week to 18 months, with an average of six months between the discovery of metastases and death [10], revealing dismal outcomes even with advanced cancer care.

Patients with urological metastases to the penis had a median cancer-specific survival time of 18 months, while those with nonurological metastases had a median cancer-specific survival time of 11 months, according to a systematic review by Cocci et al. [11]. On the prognosis of men with penile metastasis and malignant priapism, the median cancer-specific survival time for patients with urological cancer was 30 months. In comparison, the median cancer-specific survival time for individuals with nonurological cancer was 15 months [11]. The prognosis for patients with secondary penile malignancies is generally poor, with survival often reported to be less than 18 months. However, the prognosis varies significantly based on individual patient factors, including comorbidities and treatment response.

This case highlights the aggressive nature of metastatic priapism and rapid disease progression once penile metastasis has been identified. The patient succumbed to the disease within three months of diagnosis. Despite its dismal prognosis, sometimes aggressive surgical treatment is required for palliation of intractable symptoms to improve the quality of life, though it would not offer additional survival benefits. Although a few similar cases have been reported in the literature, each new case contributes valuable insights into the clinical presentation, diagnostic approaches, and management strategies for this rare condition. Documenting such cases enhances the medical community's understanding and can aid in formulating better diagnostic and therapeutic protocols.

Given the poor prognosis associated with malignant priapism, future research should focus on improving early detection methods and exploring novel therapeutic strategies. Multidisciplinary collaboration between urologists, oncologists, and radiologists is essential to optimize patient care. Additionally, there is a need for larger-scale studies that can provide more definitive guidelines for managing penile metastases and malignant priapism. Raising awareness about this rare manifestation of advanced malignancy may lead to earlier recognition and timely intervention, ultimately improving patient outcomes.

Learning points

Malignant priapism is a rare and ominous sign. It indicates widespread disease and portends a poorer prognosis. Multimodal imaging and histopathology are crucial for diagnosis. Management is mainly palliative. Though survival is often less than six months, penile amputation is warranted for palliation of intractable symptoms.

## Conclusions

Metastatic priapism is a rare but serious condition that requires prompt recognition, as it often indicates advanced disease. While imaging may give preliminary information, a conclusive diagnosis requires histological confirmation via biopsy or fine-needle aspiration. Penile involvement should be considered in individuals with a known underlying cancer if symptoms such as priapism, penile masses, or voiding difficulties occur. Aggressive surgical treatment is sometimes warranted for the palliation of intractable symptoms. Despite rigorous multimodal treatment, including systemic therapy and palliative care, the prognosis is still poor.
